# Do players with superior physiological attributes outwork their less-conditioned counterparts? A study in Gaelic football

**DOI:** 10.5114/biolsport.2024.129479

**Published:** 2023-07-24

**Authors:** Lorcan S. Daly, Ciarán Ó. Catháin, David T. Kelly

**Affiliations:** 1 Department of Sport and Health Sciences, Technological University of the Shannon, Ireland; 2 SHE Research group, Technological University of the Shannon, Ireland; 3 Department of Physical Education and Sport Sciences, University of Limerick, Ireland; 4 Sport and Human Research Centre, University of Limerick, Ireland

**Keywords:** Physical fitness, Aerobic capacity, Team Sport, Neuromuscular, External loads, GPS

## Abstract

This study investigated the association of physiological attributes with in-game workload measures during competitive Gaelic football match-play. Fifty-two male developmental level Gaelic football players (mean ± SD; age: 22.9 ± 3.8 years) underwent measurements of anthropometric characteristics, running speed, muscular strength and power, blood lactate (BLa), running economy and aerobic capacity during two separate testing visits. Global Positioning System units (18-Hz) were used to record players in-game workloads during a competitive match 1-week following the baseline physiological assessments. Results indicated that players body fat percentage, drop jump height (DJ) and running velocity at 4 mmol · L^−1^ BLa were significantly associated with the number of high-speed runs completed (Adjusted R^2^ 26.8% to 39.5%; *p* < 0.05) while 20 m running speed, running velocity at 2 mmol · L^−1^ BLa and DJ were significantly associated with the number of accelerations completed (Adjusted R^2^ 17.2% to 22.0%; *p* < 0.05) during match-play. Additionally, aerobic capacity and body fat percentage were significantly associated with total distance (Adjusted R^2^ 14.4% to 22.4%; *p* < 0.05) while body fat percentage, DJ and 20 m running speed were significantly associated with high-speed distance (Adjusted R^2^ 17.8% to 22.0%; *p* < 0.05). Players were also divided into higher-standard and lower-standard groups using a median split of these physiological attributes. Players in the higher-standard groups completed significantly more high-speed runs and accelerations and covered significantly larger total and high-speed distances (+10.4% to +36.8%; ES = 0.67 to 0.88; p < 0.05) when compared to the lower-standard groups. This study demonstrates that superior levels of physical conditioning are associated with larger in-game workloads during Gaelic football match-play.

## INTRODUCTION

Gaelic football is a field-based team sport predominantly characterised by low to moderate intensity activity interspersed with critical bouts of high-intensity actions, which can often influence a games result [1, 2]. To support these demands, a large aerobic capacity, well-developed blood lactate responses and efficient running economy (RE) may be necessary to generate and maintain the considerable workloads (≈ 100 to 130 m · min^−1^) observed during match-play [1, 2]. On the other hand, neuromuscular-related performance characteristics such as short distance running speed, power and relative strength likely underpin the performance of numerous high-speed running and power-based tasks [2, 3]. Notwithstanding the significant tactical organisation and technical skill proficiency necessary for a team sport such as Gaelic football, possessing the physical capacity to undertake a greater volume and intensity of work than the opposition is suggested to be a key requisite to successful match-play [2, 4, 5].

Whilst components of fitness are anecdotally thought to influence players in-game workloads during Gaelic football match-play [1, 3], empirical data exploring these interactions are limited. In contrast, a body of research exists assessing relationships between physical conditioning, playing standard, coaches’ perceptions of performance and in-game workload measures in team sports similar to Gaelic football [6–8]. For instance, evidence suggests that players with superior levels of aerobic-based performance attributes undertake more sprints and accelerations, cover larger total and high-speed distances and participate in more ball involvements than their less aerobically proficient counterparts in rugby union [6] and soccer [8, 9]. Although this data does not currently exist within Gaelic football, the ability to frequently express high levels of power, speed and changes of pace/direction during match-play necessitates the rapid regeneration of anaerobic substrates [10] which is a process heavily reliant on aerobic metabolism [11, 12]. In Gaelic football, inter-county players (national level; tier 3) are reported to exhibit significantly higher estimated ${\dot{\text{V}}\text{O}}_{2\max}$ values than club players (developmental level; tier 2) [13], possibly reflecting the importance of aerobic fitness in high-level performance [4]. While it may be likely that well-developed aerobic capabilities are necessary to cope with the physiological stressors of Gaelic football match-play [1], this is yet to be investigated directly.

Similar to markers of aerobic function, body composition has also been identified as an important indicator of Gaelic football performance through its impact on players capacity to run, jump and change direction/pace [3, 14, 15]. In soccer for example, players body fat levels have been negatively associated with high-speed running performance [14]. Despite the implications of these reports, an absence of applied data in Gaelic football makes it difficult to accurately surmise the extent with which body composition and other relevant components of fitness influence the unpredictable and multivariate ingame workload demands players face during competition [1].

Given the strenuous mechanical loads players are subject to during Gaelic football match-play, such as frequent accelerations/decelerations, sharp changes of direction and landing from jumps [2, 16]; it is reasonable to assume that strength, power and running speed facilitate players capacity for work [5, 17]. In team sports comparable to Gaelic football, lower body strength, power and running speed have been positively associated with acceleration and sprint number, distances covered at varying speeds and a number of key performance indicators such as effective turnovers and ball possessions during match-play [5, 6, 9]. Supporting the relevance of these markers in a team sport performance-specific setting, research in soccer reported that the number of high-intensity accelerations (> 3 m · s^−2^) and decelerations (< −3 m · s^−2^) recorded during elite match-play presented a significant dose-response relationship with match outcome; whereby outputs were highest during wins and lowest during draws or losses [7]. Similar findings were reported in Gaelic football specifically, where competitive workloads (total and high-speed distance [≥ 4.7 m · s^−1^]) were observed to be higher during wins or draws when compared with losses [18].

Successfully undertaking these tasks in a fast-paced contact environment necessitates the rapid application of high force levels accompanied by the repeated performance of quick and powerful muscular contractions [5]. Thus, corresponding relationships between neuromuscular performance characteristics and workloads may exist in Gaelic football, wherein players undertake large volumes of stretch shortening cycle based movements comprising high eccentric loads [7, 19]. Indeed, when comparing playing standards in Gaelic football elite level players display significantly greater vertical and broad jump scores than their sub-elite counterparts, possibly highlighting a key role of lower-body power in successful competitive performances [4].

In summary, the above studies have detailed how various components of fitness positively impact in-game workload measures, playing standard and match outcome in team sports similar to Gaelic football [5, 6, 9]. Nevertheless, this research has yet to be replicated in Gaelic football, where many considerations unique to the sport may limit the applicability of training or match-based decisions derived from data collected in other team sport codes. Consequential to this lack of research, pivotal assumptions as to the importance of different components of fitness in relation to game-specific work capacity are rooted in data from other sporting populations. As such, in order to provide objective data for coaches to design effective training programmes with the goal of increasing competitive workloads, it may be necessary to address these interactions in an ecologically valid context [19]. Therefore, this study aims to investigate the association of players components of fitness on in-game workload measures in Gaelic football.

## MATERIALS AND METHODS

### Subjects

Fifty-two male developmental level Gaelic football players currently representing a senior club level Gaelic football team volunteered to partake in this study. Players’ anthropometric, physiological and performance characteristics can be seen in table 1. Players were omitted from this study if they failed to pass a physical activity readiness questionnaire (PAR-Q) or had endured a lower body injury in the previous 8-weeks. Informed consent was obtained from each player and ethical approval was granted for this research by the Technological University of the Shannon Research Ethics Committee (code 20180501).

**TABLE 1:** Descriptive overview of players’ anthropometric, physiological and performance characteristics.

Baseline characteristics	Value
*Anthropometrics and body composition*	

Height (cm)	179.2 ± 7.6
Body mass (kg)	81.4 ± 9.0
Body fat (%)	14.5 ± 2.0

*Running speed*	

Running speed (5 m) (s)	1.1 ± 0.1
Running speed (20 m) (s)	3.1 ± 0.1

*Muscular power and reactive strength*	

DJ (cm)	33.6 ± 5.0
CT (s)	0.3 ± 0.1
DJ (RSI)	1.1 ± 0.4
CMJ (cm)	34.3 ± 5.2

*Muscular strength*	

1RM squat (kg)	107.4 ± 12.9
Relative 1RM squat (1RM/BM)	1.3 ± 0.2
1RM Hip thrust (kg)	127.9 ± 26.1
Relative 1RM hip thrust (1RM/BM)	1.6 ± 0.3

*Aerobic endurance*	

${\dot{\text{V}}\text{O}}_{2\max}$ (ml · kg^−1^ · min^−1^)	51.4 ± 7.2
Maximal heart rate (beats · m^−1^)	199.6 ± 6.5
Running velocity at LT (km · h^−1^)	10.5 ± 1.0
Running velocity at 2 mmol · L^−1^ BLa (km · h^−1^)	9.7 ± 1.6
Running velocity at 4 mmol · L^−1^ BLa (km · h^−1^)	13.2 ± 1.2

Abbreviations; DJ: drop jump, ${\dot{\text{V}}\text{O}}_{2\max}$: maximal aerobic capacity, LT: lactate threshold, 1RM: One repetition maximum, RSI: reactive strength index, BM: body mass, BLa: blood lactate, CT: contact time.

### Experimental Outline

**FIG. 1:**
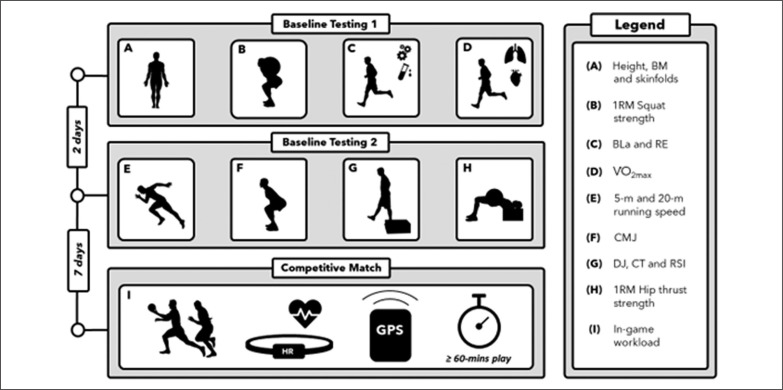
Schematic overview of study methodology.

Previous research [5, 6, 9] and the physical demands of Gaelic football match-play [2, 19] guided the selection of physical conditioning measures to be assessed during baseline testing. The testing procedures were explained to the players during a familiarization session. During visit-1, players’ anthropometric characteristics (body mass, height and body fat percentage (body fat [%]) one-repetition maximum (1RM) relative squat strength, blood lactate concentrations (BLa) (lactate threshold, running validity at 2 and 4 mmol · L^−1^), RE and maximal aerobic capacity (${\dot{\text{V}}\text{O}}_{2\max}$) were assessed. Players’ countermovement jump height (CMJ), drop jump height (DJ), contact time (CT), reactive strength index (RSI), 5 m and 20 m running speed (running speed [5 m] and [20 m]) and 1RM relative hip thrust strength were measured during the second visit to our lab. Both testing sessions were completed at the same time of day. Players were instructed to arrive hydrated and well rested. One week after the baseline testing, players’ in-game workload measures were recorded during a competitive match using global positioning system (GPS) units (Figure 1). To facilitate a high level of ecological validity, a maximum of four players were tested per game. Data was collected in 18 competitive matches spanning 2 seasons and players were recruited from 5 different teams.

### Procedures

Body mass and height were measured to the nearest 0.1 cm and 0.1 kg respectively, using a portable scales and stadiometer (Seca 707 Scales, Hamburg, Germany). Players’ skinfold thickness was measured at seven anatomical sites using a skinfold callipers (Baty, UK) as described in previous methods [19]. Here, three measurements for each skinfold on the right side of the body were obtained to the nearest 0.2 mm using the International Society for the Advancement of Kinanthropometry (ISAK) protocols [19]. Pilot testing was undertaken in order to verify the reliability of the anthropometrical measurements performed as the investigator was not ISAK accredited [20, 21]. Following intra-rater reliability assessments, the technical error of measurement of 4 repeated skinfold trials was lower than 5%, which is in line with recommendations [20]. To calculate players’ body fat (%), the equation of Withers and colleagues [22] (% BF = 495/(1.0988 − 0.0004 × [sum of seven skinfolds]) − 450) was used. This equation has previously demonstrated the low bias and high agreement (r = 0.88) with criterion dual energy absorptiometry (DEXA) measurements [23].

Players’ 1 repetition maximum (1RM) squat values were assessed using the incremental protocol described previously by Baechle and Earle [24]. Briefly, players completed a general 10-minute warm up including stationary cycling, dynamic stretching of the lower body and 10 repetitions of an empty Olympic bar (20 kg). After 2-minutes rest, an incremental warm up of 5–8 repetitions at 40% to 60% of their estimated 1RM (E1RM) followed by 3–5 repetitions at 60% to 80% of E1RM with 3–5 minutes rest between sets. Next, the lead researcher issued 1–10 kg weight increments for single repetition attempts. The players rested 3–5 minutes following each attempt and repeated as necessary until a 1RM was established. Players’ 1RM was recorded as the greatest resistance (kg) lifted successfully through a full range of motion as assessed by the lead investigator. Players’ relative strength measures were calculated by dividing 1RM values (kg) by their body mass (kg).

Players’ ${\dot{\text{V}}\text{O}}_{2\max}$ and RE were measured using a Moxus metabolic cart (AEI Technologies, PA, USA) on a motorized treadmill (Quasar, HP Cosmos, Germany) using an incremental incline ramp protocol [3, 25]. Subsequent to a 3-minute warm-up at 8km · h^−1^ on a 1% gradient, the speed of the treadmill incrementally increased by 1 km · h^−1^ every 3-minutes until the players’ blood lactate concentrations reached 4 mmol · L^−1^ or higher. To collect lactate samples, the base of the earlobe was first wiped with an alcohol wipe and allowed to dry, before being pierced with a lancet (AccuChek; Softclix, Roche, Germany). The first drop of blood was then swabbed away with another alcohol wipe and pressure was then applied with the thumb and forefinger to draw a 5-μl capillary blood sample. The sample was automatically aspirated (via capillary action) into an enzyme-coated electrode test strip and analysed using a portable amperometric microvolume lactate analyser (Lactate Pro 2, Arkray, Japan) to determine whole BLa concentration. In order to collect the sample, players stepped to the side and straddled the treadmill for approximately 20 seconds following completion of the previous stage, and the speed and/or incline was changed accordingly before the subsequent stage. The lactate analyser used has previously demonstrated high levels of validity (intraclass correlation coefficient [ICC] = 0.87) and reliability (ICC = 0.99) [26]. Applying methods previously described elsewhere [3, 25], plots of BLa, ${\dot{\text{V}}\text{O}}_{2}$ and running speed were generated with an exponential trendline and provided to two independent reviews. Lactate threshold (LT) was identified as the first sustained increase in BLa above the baseline values. Markers of 2 and 4 mmol · L^−1^ were also identified on these plots. Once 4 mmol · L^−1^ or greater was recorded, a constant speed of 10 km · h^−1^ was set on a 4% gradient, increasing the gradient by 1% each minute until volitional exhaustion [25, 27]. Players’ heart rate (HR) was measured using a watch and chest strap (Polar Vantage, Polar Electro, Finland) at the end of each stage. During the test, expired CO_2_, expired O_2_, ventilatory volume/rate, HR and rate of perceived exertion were continuously monitored. RE and ${\dot{\text{V}}\text{O}}_{2\max}$ were reported in ml · kg^−1^ · min^−1^ using the average of the final two 30 s values [25].

Players’ running speed was recorded over 5 m and 20 m using photoelectric cells (Brower Timing Systems, UT, USA). These photoelectric cells have been reported as valid (standard error of measurement = 0.1 s) and reliable (coefficient of variation = 0.1%) [28]. The players were instructed to start by placing forward their dominant foot on a mark 50 cm behind the starting line. Timing gates were placed at the starting line, 5 m line and the 20 m line. Players completed three runs, with the best time recorded as their result. Players rested 4–5 minutes between each trial.

Players’ CMJ and DJ were evaluated using a photoelectric optical device (Optojump, Microgate, Italy). This measurement system has previously displayed low random errors (± 2.8 cm) and coefficients of variation (2.7%) [29]. Players performed the CMJ whilst standing on a standardised surface between two photoelectric measurement bars with hands placed on their hips. Next, a countermovement action was undertaken by the players using self-selected ankle, knee and hip flexion angles before jumping vertically as high as possible. When performing the DJ, players began the jump standing on a 30 cm high step with their hands placed on their hips before stepping off and immediately jumping vertically as high as possible when contacting the ground. Here, players were instructed to jump to a maximal height while simultaneously minimising ground contact time. The players’ 1RM hip thrust was assessed using the same incremental protocol as the squat test [24].

Players’ in-game workloads were recorded using 18 Hz GPS units (Apex, STATSports, UK). This model of GPS unit has previously demonstrated good levels of validity (Bias < 5.0%) [30]. Players were fitted with an appropriately sized vest to hold the GPS receiver. Each GPS unit was powered on 15-minutes before throw-in and inserted into a slot towards the top of the fitted vest sitting in the upper back. A HR telemetry system (Polar Vantage, Polar Electro, Finland) with an accompanying strap was placed around the chest to collect HR data. The workload and HR data were then extracted from the GPS devices and downloaded using the STATSports analysis platform [19]. Total distance (m), total accelerations (*n*) (≥ 3 m · s^−2^), total high-speed runs (*n*) (≥ 5 · 5 m · s^−1^), and high-speed distance (≥ 5.5 m · s^−1^) were the metrics used to quantify in-game workloads in the current analysis. These metrics were specifically selected as they have been commonly used in the literature and may subsequently help translate current results into applied and research settings [2, 16, 31, 32].

### Statistical Analyses

Descriptive statistics using means and standard deviations (± SD) were calculated for all anthropometric characteristics, components of fitness and in-game workload measures. All data were normally distributed as assessed by Shapiro-wilk tests. Standard multiple regression analysis was performed to examine possible relationships between components of fitness and in-game workload measures [33]. These tests were selected in order to provide a statistical summary of the associations (or lack thereof) between the various independent variables whilst accounting for potential between-variable interdependence [33]. An *a priori* power analysis was conducted to estimate the sample requirements for the multiple regression analysis using G*Power (version 3.1.9.7). Power was set at 0.95 in conjunction with a significance level of 0.05 and an effect size (ES) of 0.3, which yielded a minimum sample size of 38 players [33]. Additional players were requited to account for possible drop out. A median split divided players into higher-standard (HS) and lower-standard (LS) groups for each component of fitness significantly contributing to the multiple regression analysis, similar to previous work in the domain [5, 34]. Independent samples t tests and Cohen’s ES statistic were used to examine differences between-group differences. Effect sizes of > 0.20, 0.20–0.60, 0.61–1.19, and > 1.20 were considered trivial, small, moderate, and large respectively [35]. Data were analysed using Statistical Package for Social Sciences (SPSS Version 27, Chicago, USA). Statistical significance was accepted at an alpha level of *p* < 0.05.

## RESULTS

**FIG. 2:**
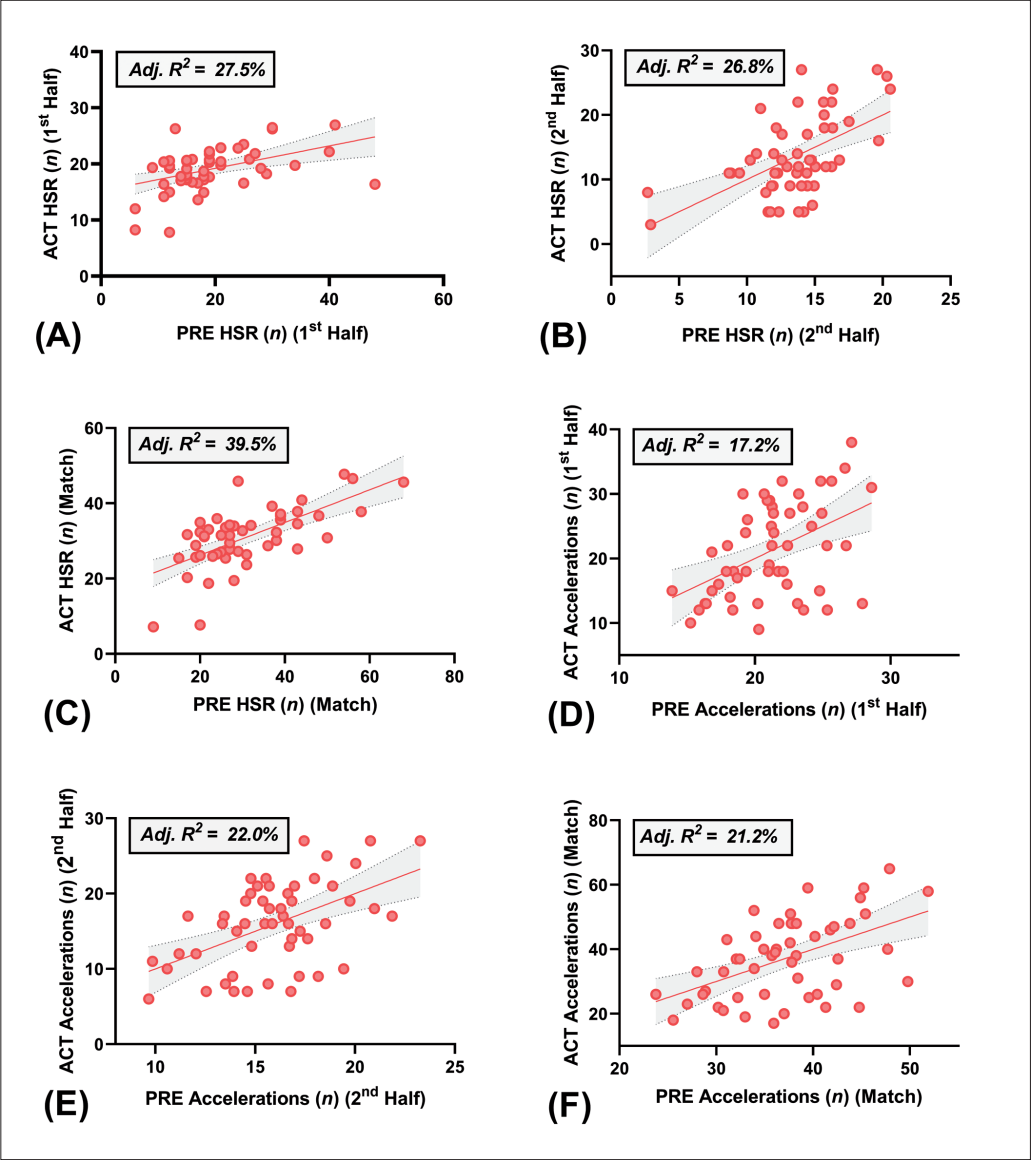
Plot of actual (ACT) vs. predicted (PRE) values for the number of **(A)** 1^st^ half high-speed runs (HSR), **(B)** 2^nd^ half HSR, **(C)** full match HSR, **(D)** 1^st^ half accelerations, **(E)** 2^nd^ half accelerations and **(F)** full match accelerations completed during match-play. Dependant variables for total HSR (*n*) (≥ 5.5 m · s^−1^): body fat (%), drop jump and running velocity at 4 mmol · L^−1^. Dependant variables for total accelerations (*n*) (≥ 3 m · s ^−2^): running speed (20 m), running velocity at 2 mmol · L^−1^ and DJ). Shading represents 95% confidence intervals.

**FIG. 3:**
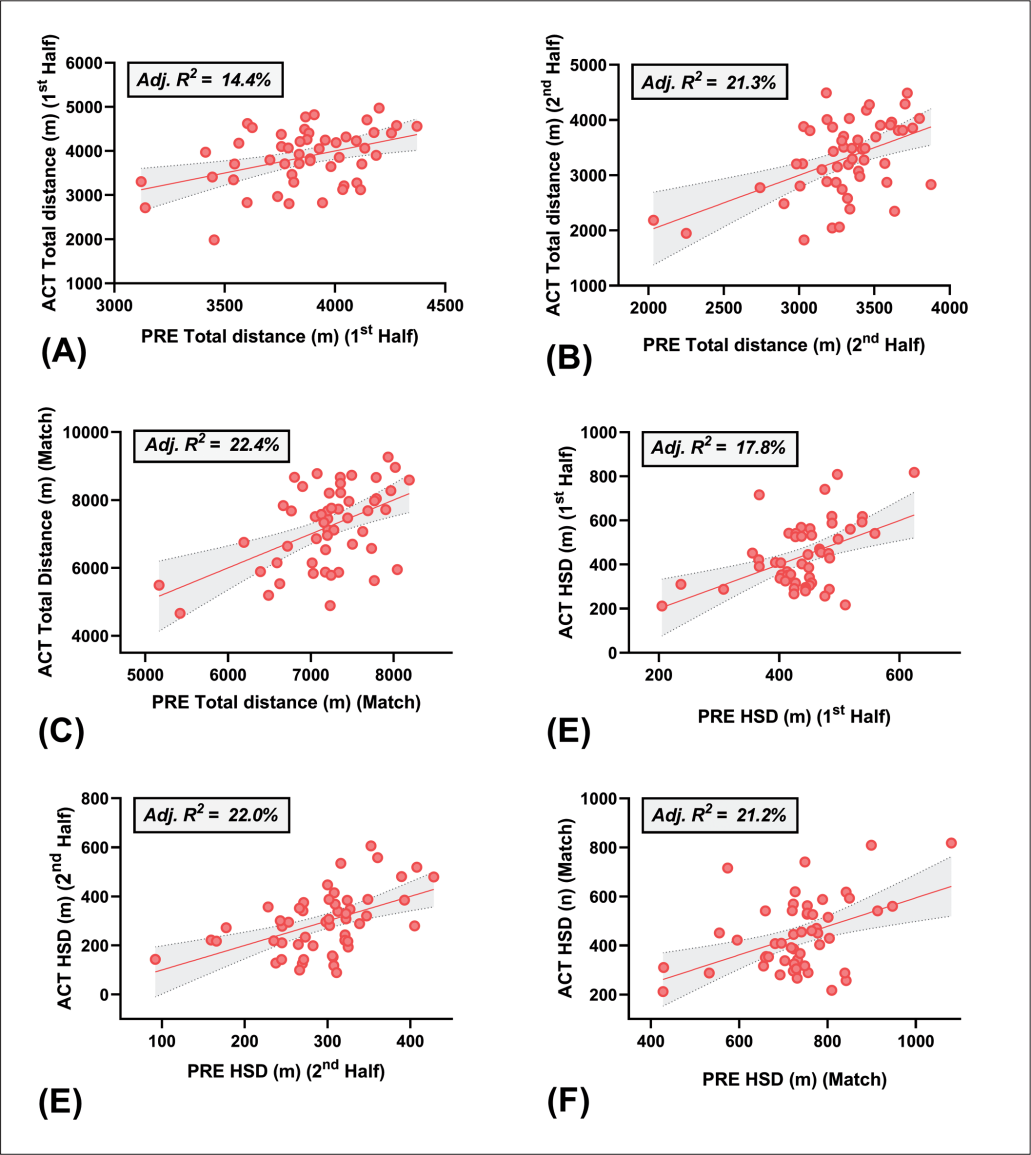
Plot of actual (ACT) vs. predicted (PRE) values for **(A)** 1^st^ half distance, **(B)** 2^nd^ half distance, **(C)** full match distance, **(D)** 1^st^ half high-speed distance (HSD), **(E)** 2^nd^ half HSD and **(F)** full match HSD covered during match-play. Total distance (m) dependant variables: ${\dot{\text{V}}\text{O}}_{2\max}$ and body fat (%). HSD (≥ 5.5 m · s ^−1^) (m) dependent variables: body fat (%), drop jump and 20 m running speed. Shading represents 95% confidence intervals.

Results of the multiple regression analysis are summarised in table 2 and the plots of actual versus predicted residuals are depicted in figure 2 and 3.

**TABLE 2:** Summary of Multiple regression analysis.

Model predictors	Dependant variable	Adj. R^2^ (%)	p value
Body fat (%), DJ and running velocity at 4 mmol · L^−1^	HSR (*n*) (≥ 5.5 m · s^−1^) (1^st^ half)	27.5	< 0.001
	HSR (*n*) (≥ 5.5 m · s^−1^) (2^nd^ half)	26.8	< 0.001
	HSR (*n*) (≥ 5.5 m · s^−1^) (Match)	39.8	< 0.001

Relative squat strength and DJ	Total accelerations (*n*) (≥ 3 m · s ^−2^) (1^st^ half)	17.2	0.007
	Total accelerations (*n*) (≥ 3 m · s ^−2^) (2^nd^ half)	22.0	0.002
	Total accelerations (*n*) (≥ 3 m · s ^−2^) (Match)	21.2	0.002

${\dot{\text{V}}\text{O}}_{2\max}$ and body fat (%)	Total distance covered (m) (1^st^ half)	14.4	0.009
	Total distance covered (m) (2^nd^ half)	21.3	0.001
	Total distance covered (m) (Match)	22.4	0.001

Running speed (20 m), running velocity at 2 mmol · L^−1^ and DJ	HSD (m) (≥ 5.5 m · s^−1^) (1^st^ half)	17.8	0.006
	HSD (m) (≥ 5.5 m · s^−1^) (2^nd^ half)	21.2	0.002
	HSD (m) (≥ 5.5 m · s^−1^) (Match)	27.0	< 0.001

Abbreviations; Adj: Adjusted, DJ: drop jump, HSD: high-speed distance, HSR: high-speed runs, ${\dot{\text{V}}\text{O}}_{2\max}$: maximal aerobic capacity, 1^st^ half: first half of a match, 2^nd^ half: second half of match; Match: full match.

Table 3 shows the HS and LS descriptive variables for each component of fitness.

**TABLE 3:** Descriptive results for each higher-standard and lower-standard group.

Component of Fitness	Higher standard	Lower standard
Body fat (%)	11.5 ± 2.0	17.5 ± 4.1
${\dot{\text{V}}\text{O}}_{2\max}$ (ml · kg^−1^ · min^−1^)	57.4 ± 4.2	45.3 ± 3.8
Running velocity at 2 mmol · L^−1^ (km · h^−1^)	11.2 ± 0.8	8.2 ± 0.4
Running velocity at 4 mmol · L^−1^ (km · h^−1^)	14.4 ± 0.7	12.5 ± 0.8
Running speed (20 m) (s)	3.04 ± 0.04	3.18 ± 0.05
DJ (cm)	37.6 ± 2.5	29.7 ± 3.6

Abbreviations; DJ: drop jump, ${\dot{\text{V}}\text{O}}_{2\max}$: maximal aerobic capacity.

**FIG. 4:**
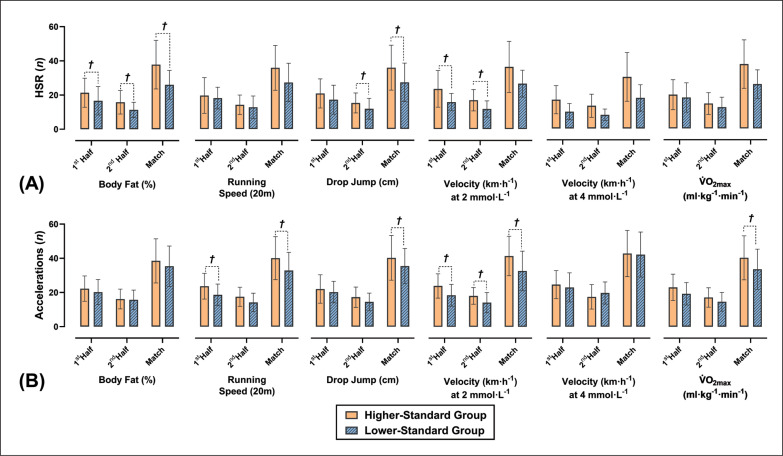
Differences in the number of accelerations **(A)** and high-speed runs (HSR) **(B)** completed during match-play between higherstandard (HS) and lower-standard (LS) component of fitness groups. † Denotes a significant difference between HS and HS groups.

The differences between HS and LS groups based off each component of fitness are shown in figure 3 and 4. When players were dichotomized into HS and LS running velocity at 2 mmol · L^−1^ groups, the HS group performed significantly more 1^st^ half accelerations (ES = 0.76; *p* = 0.008), 2^nd^ half accelerations (ES = 0.64; *p* = 0.027) and total accelerations (ES = 0.76; *p* = 0.009) than the LS group. Additionally, the HS running velocity at 2 mmol · L^−1^ group covered significantly larger 1^st^ half high-speed distance (HSD) (ES = 0.62; *p* = 0.031) and performed significantly more 1^st^ half high-speed runs (HSR) (ES = 0.88; *p* = 0.003), 2^nd^ half HSR (ES = 0.86; p = 0.003) and total HSR (ES = 0.95; *p* < 0.001) when compared to the LS group. When players were divided into HS and LS running velocity at 4 mmol · L^−1^ groups the HS group performed significantly more total HSR (ES = 0.68; *p* = 0.015) than the LS group. When players were divided into HS and LS ${\dot{\text{V}}\text{O}}_{2\max}$ groups the HS group covered significantly greater 1^st^ half distances (ES = 0.56; *p* = 0.048) and total distances (ES = 0.57; *p* = 0.045) when compared to the LS group.

When players were divided into HS and LS body fat (%) groups, the HS group covered significantly greater 2^nd^ half distances (ES = 0.75; *p* = 0.038), total distances (ES = 0.67; *p* = 0.20), 1^st^ half HSD (ES = 0.58; *p* = 0.042) and total HSD (ES = 0.59; *p* = 0.038) when compared to the LS group. Additionally, players in the HS body fat (%) group performed significantly more 2^nd^ half HSR (ES = 0.77; *p* = 0.008) and total HSR (ES = 0.71; *p* = 0.014) when compared to the LS group. Furthermore, when players were split into HS and LS 20 m running speed groups, the HS group performed significantly more accelerations during the 1^st^ half (ES = 0.74; *p* = 0.011), 2^nd^ half (ES = 0.60; *p* = 0.035) and full game (ES = 0.72; *p* = 0.013) when compared to the LS group. Finally, when players were dichotomized into HS and LS DJ groups, players in the HS group performed significantly more 2^nd^ half HSR (ES = 0.57; *p* = 0.047) than the LS group.

## DISCUSSION

**FIG. 5:**
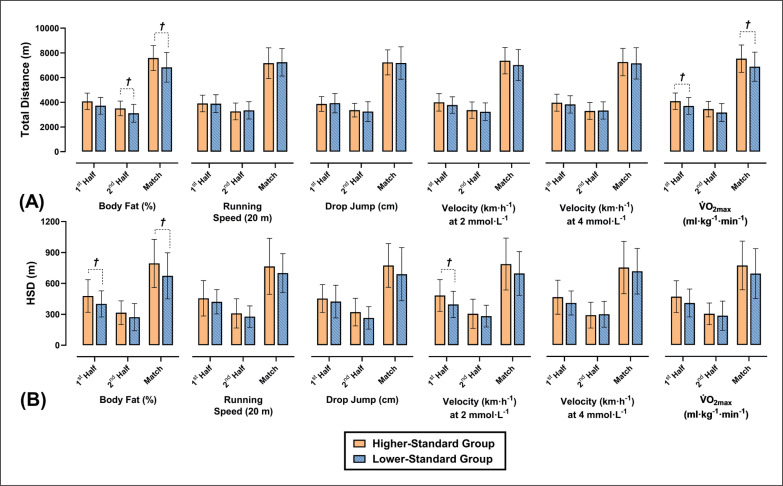
Differences in total distance covered **(A)** and high-speed distance (HSD) covered **(B)** during match-play between higherstandard (HS) and lower-standard (LS) component of fitness groups. † Denotes a significant difference between HS and HS groups.

This study aimed to investigate the association of Gaelic football players body composition and markers of physical conditioning with in-game workload measures. Some of the physiological attributes measured are the first to be reported in Gaelic football, and the markers may be used by practitioners as benchmark scores for training and profiling purposes (Table 2). Additionally, the workload data reported is similar to prior analysis in developmental Gaelic football players [2] and may be used by practitioners to structure and monitor training sessions and inform return to play protocols (figure 4 and 5). The current data demonstrates that a wide range of components of fitness significantly associate with external load measures during competitive match-play, complimenting research in similar team sports [8, 9, 34].

Similar to previous results in soccer [36], blood lactate responses were significantly associated with the number of accelerations/HSR completed and the HSD covered during competitive match-play (table 1). Capacity to perform these high-intensity tasks are often crucial for team sport performance; possibly serving as a surrogate measure of successful contests for possession, entering space during scoring opportunities, defensively tracking opposition players movements and coping with fast-paced and potentially evolving tactical requirements [2, 37, 38]. Moreover, when players were divided into HS and LS groups based on running running velocity at 2 mmol · L^−1^, the HS groups performed significantly more accelerations and HSR during match-play than the LS groups, which corresponds with previous work in ice hockey [39]. Equivalent associations were also reported in female soccer players, where lactate responses were significantly correlated with high-intensity running distances during simulated [40] and competitive match-play [36].

Other markers of aerobic function displayed comparable trends in the present analysis, whereby well-developed ${\dot{\text{V}}\text{O}}_{2\max}$ (in combination with body fat [%]) significantly contributed to a regression model associated with total distance covered during match-play. Furthermore, when players were dichotomised into groups using higher and lower ${\dot{\text{V}}\text{O}}_{2\max}$ scores, the HS ${\dot{\text{V}}\text{O}}_{2\max}$ group covered significantly greater total distances when compared to their LS group counterparts, which is consistent with work in rugby league [5]. These collective observations present evidence that aerobic capacity plays a fundamental role in the considerable running demands undertaken during team sport match-play. In line with this premise, aerobic capacity was positively associated with total distance, high speed running distance and high-intensity efforts during rugby league [5], Australian rules football [41] and soccer [9] match-play respectively. Furthermore, well-developed aerobic conditioning and blood lactate response have been reported to accelerate metabolite clearance, improve phosphocreatine resynthesis and reduce the metabolic and cardiovascular strain associated with recurrent high-intensity running [10, 25] possibly helping to explain current findings. Specifically, energy for these high-intensity actions is supplied by anaerobic metabolism resulting in lactate formation, hydrogen ion accumulation, pH reduction and the depletion of adenosine triphosphate phosphocreatine stores paralleled with inorganic phosphate increases [11, 12, 25]. In response to these disruptions, oxidative phosphorylation is a necessary process to achieve adequate recovery prior to the next bout of intense work during matchplay and this mechanism is enhanced in aerobically fitter athletes [10, 11]. Overall, many possible adaptations associated with well-developed aerobic function and/or blood lactate responses, such as increased muscle capillarization, mitochondrial volume and density and oxidative enzyme activity could have possibly contributed to the present results [3, 10, 12].

Similar to research in soccer [14], body fat percentage was negatively associated with the number of HSR and accelerations completed and the total and HSD covered. Body composition exerts an important influence on players game-specific work capacity, as excess adipose tissue can increase mechanical and metabolic strain by adding unnecessary resistance against the forces of gravity during movements such as jumping, running or changes of direction [14, 15, 23]. In addition to the energy cost of bearing a larger body mass over the course of a match, it is possible that excessive adipose tissue in Gaelic football players could impair thermoregulatory function by impeding capacity to transport and dissipate metabolically generated body heat, thereby potentially disrupting thermal balance [14, 42]. In support of this premise, it is commonly reported that elite level players possess lower body fat percentages then their sub-elite counterparts within Gaelic football [4] and other team sports [43]. Considering the potential overlap in training adaptations associated with improving body composition, blood lactate responses and aerobic conditioning; programming strategies to increase these attributes in tandem with the goal of improving players work capacity may possibly be employed with relatively minimal physiological interference [44]. That being said, the multifaceted physical demands of Gaelic football match-play imply that other components of fitness such as strength, power and running speed may also moderate players external match-play loads as has been reported previously in other team sports [5, 9, 17].

Although movement patterns during invasive team sports such as Gaelic football are predominantly low-intensity in nature, explosive stretch shortening cycle actions imposing high mechanical tension regularly occur at pivotal moments of a game [1, 2]. As a consequence of such taxing neuromuscular demands, muscular strength and power are commonly acknowledged as important attributes for team sport performance [5, 27, 45]. Indeed, measures of maximal force and power generating capacity have been previously related to in-game workload measures such as running speed and the number of sprints/accelerations completed in rugby league [5]. Our data supports these findings, whereby DJ was significantly associated with the number of HSR and accelerations completed. Therefore, coaches may select the development of players lower body power as a potentially effective means to increase high intensity running indices and the capacity to perform numerous accelerations during competitive matches. In agreement with prior work [46], the current analysis reported that players in the HS 20 m running speed group performed significantly more accelerations during match-play than their LS group counterparts. Based on this data, it is possible that the number of HSR and accelerations recorded during competitive Gaelic football match-play may be modulated by players baseline DJ and 20 m running speed values. If this is the case, it may be possible that implementing training processes to develop these performance attributes may deliver a high transfer efficacy for Gaelic football specific tasks and subsequently promote players external workloads, and ultimately performance. Of note, caution should be exercised when interpreting data such as accelerations or decelerations during match-play, given variance in approach demands (e.g., walking vs. sprinting) will greatly alter the physiological demands imposed by a change of pace, yet will be reflected in the same net increase or decrease in speed [47, 48].

The current findings provide evidence that Gaelic football players competitive running and acceleration profiles are associated with their physical conditioning attributes. Specifically, lower body power, running speed, body composition, aerobic capacity and blood lactate responses display significant associations, which is complementary to findings in other team sports [9, 36, 39]. Furthermore, when players were dichotomised into HS and LS groups based off these conditioning markers, the HS groups almost always performed more external work than the LS groups. In combination with prior research [27], these findings suggest that players with well-developed components of fitness express comparatively lower fatigue and muscle damage responses, even despite undertaking larger external loads during competition than their less physically conditioned counterparts. This data may improve coaches understanding of the complex interactions between physical conditioning and in-game workload measures, thereby providing relevant guidance for effective training programme design intent on increasing sport-specific work capacity. Despite promising findings as to the role of physical conditioning in the determination of game-based workloads, this data is most relevant to the population studied and limitations may exist applying this research in female players or in other team sport athletes. Finally, future research assessing relationships between players physical conditioning attitudes and coaches subjective scoring of match performance would provide beneficial information for a more sport-specific outlook when viewed in conjunction with the current work. Whilst the current findings demonstrate that various physiological attributes coincide with superior match-specific work capacity, it is difficult to surmise whether improvements in these physiological parameters would elicit meaningful increases in these outcomes. To the best of the authors knowledge, this has yet to be examined in any team sport population, as the contemporary work in the area exclusively employs cross-sectional and observational study designs [6, 8, 9, 39]. Hence, as highlighted by Steel and colleagues [49], causality may not be assumed from the present work or many previous analyses, and future research should seek to investigate if the further development of physical conditioning measures provides additional benefits for increasing in-game workloads. Furthermore, if the links are indeed reported to be causative, it would be useful for practitioners to understand: (1) does the magnitude of enhanced responses reflect the magnitude of physiological improvements? or (2) is there a ceiling effect, whereby increases in physical conditioning beyond a certain point may present diminishing returns? Critically, studies utilising appropriate methods to infer causation are needed to explore this complex and mediator-entangled area [49, 50].

## CONCLUSIONS

In this study, Gaelic football players with superior neuromuscular and aerobic characteristics demonstrated larger workloads during competitive match-play. Our results reflect the multifaceted physical and metabolic loads players face during match-play, highlighting a significant association between competitive workload measures and body composition, running speed, muscular power, reactive strength and aerobic conditioning. The data herein provide potentially useful information for coaches and practitioners who seek to increase players’ external loads during competition. An important avenue for future exploration is to characterise the dose-response relationship between physical conditioning and workload using controlled inference methods.

### Practical applications

The numerous physical, tactical and technical components that govern successful Gaelic football match-play make effective preparation for the sport’s demands a complex challenge. Whilst directionally associated, physical conditioning appears to represent a clouded predictor of in-game workload at best, and this finding emphasises the premise that many moderators collectively bear an influence upon match-play locomotor profiles (e.g., score line, opposition, technical-tactical and psycho-social factors). Nevertheless, the novel findings presented support the contention that developing players metabolic, mechanical and neuromuscular properties may translate into the performance of larger total and high intensity external loads. Therefore, adopting periodised concurrent training approaches that appropriately integrate strength and/or power training with endurance training may improve on-field work capacity [3, 17, 50].
